# μ-4,4′-Bipyridine-κ^2^ *N*:*N*′-bis­[bis­(2-chloro­benzoato-κ^2^ *O*,*O*′)lead(II)]

**DOI:** 10.1107/S1600536811022422

**Published:** 2011-06-18

**Authors:** Tian-Jun Feng

**Affiliations:** a College of Mathematics, Physics and Software Engineering, Lanzhou Jiaotong University, Lanzhou 730070, People’s Republic of China

## Abstract

In the title dinuclear complex, [Pb_2_(C_7_H_4_ClO_2_)_4_(C_10_H_8_N_2_)], the Pb^II^ atom is five-coordinated by four carboxyl­ate O atoms from two 2-chloro­benzoate ligands and one N atom from a bridging 4,4′-bipyridine (4,4′-bpy) ligand, displaying a hemi-directed coordination. The 4,4′-bpy ligand has an inversion center at the mid-point of the central C—C bond. The empty side of the metal ion is capped by two carboxyl­ate O atoms from a neighboring mol­ecule, with weak Pb⋯O contacts [Pb⋯O = 3.069 (2) and 3.071 (3) Å]. The crystal structure is stabilized by C—H⋯O hydrogen bonds and π–π stacking inter­actions between the benzene and pyridine rings [centroid–centroid distance = 3.749 (3) Å].

## Related literature

For general background to 2-chloro­benzoate complexes, see: Gomez & Corbella (2009 ▶); Motokawa *et al.* (2010 ▶). For general background to 4,4′-bipyridine complexes, see: Biradha *et al.* (2006 ▶). For hemi- and holo-directed geometries of lead(II) complexes, see: Shimoni-Livny *et al.* (1998 ▶).
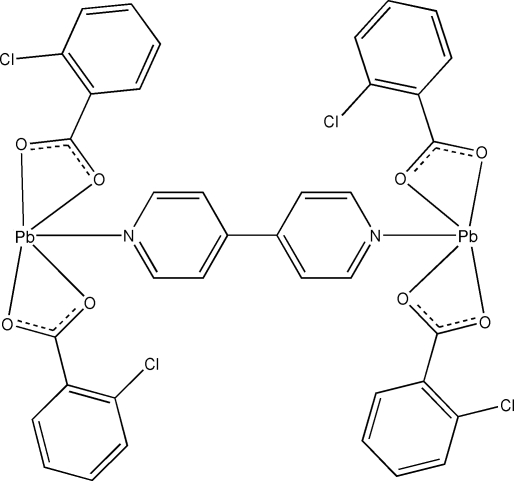


## Experimental

### 

#### Crystal data

[Pb_2_(C_7_H_4_ClO_2_)_4_(C_10_H_8_N_2_)]
*M* *_r_* = 1192.79
Orthorhombic, 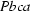

*a* = 21.9370 (13) Å
*b* = 7.4569 (5) Å
*c* = 23.2379 (14) Å
*V* = 3801.3 (4) Å^3^
*Z* = 4
Mo *K*α radiation
μ = 9.18 mm^−1^
*T* = 296 K
0.30 × 0.28 × 0.22 mm

#### Data collection

Bruker APEXII CCD diffractometer
Absorption correction: multi-scan (*SADABS*; Sheldrick, 1996 ▶) *T*_min_ = 0.092, *T*_max_ = 0.145
15375 measured reflections
3398 independent reflections
2712 reflections with *I* > 2σ(*I*)
*R*_int_ = 0.032

#### Refinement

*R*[*F* ^2^ > 2σ(*F* ^2^)] = 0.026
*wR*(*F* ^2^) = 0.081
*S* = 1.09
3398 reflections
244 parameters
H-atom parameters constrained
Δρ_max_ = 0.84 e Å^−3^
Δρ_min_ = −0.53 e Å^−3^

### 

Data collection: *APEX2* (Bruker, 2007 ▶); cell refinement: *SAINT* (Bruker, 2007 ▶); data reduction: *SAINT*; program(s) used to solve structure: *SHELXS97* (Sheldrick, 2008 ▶); program(s) used to refine structure: *SHELXL97* (Sheldrick, 2008 ▶); molecular graphics: *SHELXTL* (Sheldrick, 2008 ▶) and *Mercury* (Macrae *et al.*, 2006 ▶); software used to prepare material for publication: *SHELXTL*.

## Supplementary Material

Crystal structure: contains datablock(s) I, global. DOI: 10.1107/S1600536811022422/hy2436sup1.cif

Structure factors: contains datablock(s) I. DOI: 10.1107/S1600536811022422/hy2436Isup2.hkl

Additional supplementary materials:  crystallographic information; 3D view; checkCIF report

## Figures and Tables

**Table 1:** Hydrogen-bond geometry (Å, °)

*D*—H⋯*A*	*D*—H	H⋯*A*	*D*⋯*A*	*D*—H⋯*A*
C15—H15⋯O1^i^	0.93	2.40	3.239 (7)	150
C16—H16⋯O2^ii^	0.93	2.55	3.361 (6)	146
C19—H19⋯O4	0.93	2.39	3.026 (5)	125

Symmetry codes: (i) 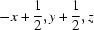; (ii) 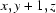.

**Fig. 1.**
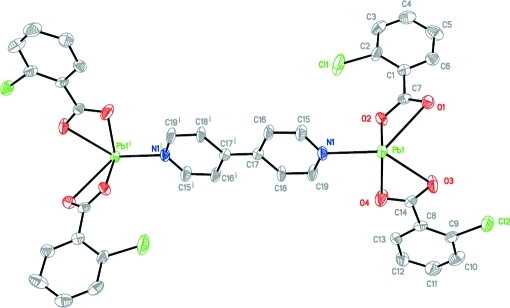
The molecular structure of the title compound, showing 30% probability displacement ellipsoids. H atoms were omitted for clarity. [Symmetry code: (i) -x, 3-y, -z.]

**Fig. 2.**
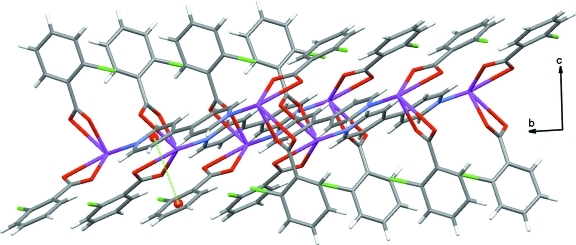
A packing view of the title compound. π–π interactions are shown as dashed lines.

## supplementary crystallographic information

### Comment

In the structural investigation of 2-chlorobenzoate complexes, it has been found that 2-chlorobenzoic acid (2-Hcbz) functions as a multidentate ligand with versatile binding and coordination modes (Gomez & Corbella, 2009; Motokawa *et al.*, 2010). As is well known, 4,4'-bipyridine (4,4'-bpy) ligand can act in bidentate bridging or monodentate terminal modes (Biradha *et al.*, 2006). In this paper, we report the crystal structure of the title compound, a new Pb(II) complex obtained by the reaction of 2-Hcbz, 4,4'-bpy and lead(II) acetate in an alkaline aqueous solution.

As depicted in Fig. 1, the Pb^II^ atom is coordinated by four O atoms from two 2-cbz ligands and one N atom from a µ-4,4'-bpy ligand. The coordination environment of the Pb^II^ atom is hemidirected. The empty sapce around the metal ion is filled by the stereochemically active 6S^2^ electron pair (Shimoni-Livny *et al.*, 1998) and two Pb···O contacts [Pb1···O1^i^ = 3.069 (2), Pb1···O3^i^ = 3.071 (3) Å. Symmetry code: (i) 1/2-*x*, 1/2+*y*, *z*]. The µ-4,4'-bpy ligand, having an inversion center at the mid-point of the central C—C bond, bridges two Pb atoms, with a Pb1···Pb1^ii^ distance of 12.073 (3) Å [symmetry code: (ii) -*x*, 3-*y*, -*z*]. The interactions of the structural components are governed by Pb···O contacts, C—H···O hydrogen bonds (Table 1) and π–π stacking interactions. The centroid–centroid distance between the benzene ring of a 2-cbz ligand and the pyridine ring of a 4,4'-bpy at (*x*, -1+*y*, *z*) is 3.749 (3) Å (Fig. 2).

### Experimental

A mixture of lead acetate (1 mmol, 0.325 g), 2-Hcbz (1 mmol, 0.156 g), 4,4'-bpy (1 mmol, 0.156 g), NaOH (1.5 mmol, 0.06 g) and H_2_O (12 ml) was placed in a 23 ml Teflon-lined reactor, which was heated at 433 K for three days and then cooled to room temperature at a rate of 10 K h^-1^. The colorless crystals obtained were washed with water and dried in air.

### Refinement

H atoms were placed at calculated positions and treated as riding atoms, with C—H = 0.93 Å and with *U*_iso_(H) = 1.2*U*_eq_(C). The highest peak in final difference map is located 0.85 Å from Pb1 and the deepest hole is located 0.40 Å from Cl2.

### Crystal data

**Table:**

[Pb_2_(C_7_H_4_ClO_2_)_4_(C_10_H_8_N_2_)]	*F*(000) = 2248
*M**_r_* = 1192.79	*D*_x_ = 2.084 Mg m^−^^3^
Orthorhombic, *P**b**c**a*	Mo *K*α radiation, λ = 0.71073 Å
Hall symbol: -P 2ac 2ab	Cell parameters from 5300 reflections
*a* = 21.9370 (13) Å	θ = 1.3–28.0°
*b* = 7.4569 (5) Å	µ = 9.18 mm^−^^1^
*c* = 23.2379 (14) Å	*T* = 296 K
*V* = 3801.3 (4) Å^3^	Block, colorless
*Z* = 4	0.30 × 0.28 × 0.22 mm

### Data collection

**Table:**

Bruker APEXII CCD diffractometer	3398 independent reflections
Radiation source: fine-focus sealed tube	2712 reflections with *I* > 2σ(*I*)
graphite	*R*_int_ = 0.032
φ and ω scans	θ_max_ = 25.2°, θ_min_ = 1.8°
Absorption correction: multi-scan (*SADABS*; Sheldrick, 1996)	*h* = −25→26
*T*_min_ = 0.092, *T*_max_ = 0.145	*k* = −8→8
15375 measured reflections	*l* = −27→19

### Refinement

**Table:**

Refinement on *F*^2^	Primary atom site location: structure-invariant direct methods
Least-squares matrix: full	Secondary atom site location: difference Fourier map
*R*[*F*^2^ > 2σ(*F*^2^)] = 0.026	Hydrogen site location: inferred from neighbouring sites
*wR*(*F*^2^) = 0.081	H-atom parameters constrained
*S* = 1.09	*w* = 1/[σ^2^(*F*_o_^2^) + (0.0403*P*)^2^ + 6.2959*P*] where *P* = (*F*_o_^2^ + 2*F*_c_^2^)/3
3398 reflections	(Δ/σ)_max_ = 0.001
244 parameters	Δρ_max_ = 0.84 e Å^−^^3^
0 restraints	Δρ_min_ = −0.53 e Å^−^^3^

### Fractional atomic coordinates and isotropic or equivalent isotropic displacement parameters (Å^2^)

**Table:**

	*x*	*y*	*z*	*U*_iso_*/*U*_eq_	
C1	0.1794 (3)	0.7076 (9)	−0.1258 (3)	0.0427 (15)	
C2	0.1528 (3)	0.8251 (10)	−0.1646 (3)	0.0552 (18)	
C3	0.1478 (4)	0.7786 (12)	−0.2219 (3)	0.068 (2)	
H3	0.1289	0.8564	−0.2476	0.082*	
C4	0.1705 (4)	0.6187 (13)	−0.2411 (3)	0.070 (2)	
H4	0.1692	0.5922	−0.2802	0.084*	
C5	0.1949 (4)	0.4974 (16)	−0.2036 (4)	0.082 (3)	
H5	0.2071	0.3840	−0.2157	0.098*	
C6	0.2009 (3)	0.5513 (11)	−0.1462 (3)	0.0555 (19)	
H6	0.2208	0.4748	−0.1208	0.067*	
C7	0.1912 (3)	0.7545 (9)	−0.0634 (3)	0.0405 (14)	
C8	0.0956 (3)	0.5487 (8)	0.1402 (2)	0.0389 (14)	
C9	0.1121 (3)	0.3828 (10)	0.1618 (3)	0.0517 (17)	
C10	0.0715 (4)	0.2756 (12)	0.1901 (3)	0.075 (2)	
H10	0.0851	0.1680	0.2058	0.090*	
C11	0.0125 (4)	0.3208 (13)	0.1958 (4)	0.080 (3)	
H11	−0.0153	0.2422	0.2124	0.096*	
C12	−0.0058 (3)	0.4903 (11)	0.1761 (3)	0.060 (2)	
H12	−0.0458	0.5282	0.1815	0.071*	
C13	0.0351 (3)	0.5999 (10)	0.1490 (3)	0.0498 (17)	
H13	0.0222	0.7116	0.1360	0.060*	
C14	0.1369 (3)	0.6803 (9)	0.1091 (2)	0.0421 (15)	
C15	0.1235 (3)	1.3106 (9)	−0.0034 (3)	0.059 (2)	
H15	0.1630	1.3251	−0.0172	0.071*	
C16	0.0814 (3)	1.4422 (9)	−0.0149 (4)	0.060 (2)	
H16	0.0930	1.5416	−0.0364	0.072*	
C17	0.0229 (2)	1.4299 (7)	0.0047 (3)	0.0343 (13)	
C18	0.0088 (3)	1.2696 (9)	0.0332 (3)	0.0503 (18)	
H18	−0.0308	1.2478	0.0457	0.060*	
C19	0.0541 (3)	1.1442 (9)	0.0427 (3)	0.0547 (18)	
H19	0.0443	1.0388	0.0620	0.066*	
Cl1	0.12558 (15)	1.0350 (3)	−0.14483 (12)	0.1014 (9)	
Cl2	0.18725 (7)	0.3008 (3)	0.15571 (9)	0.0618 (5)	
N1	0.1109 (2)	1.1684 (7)	0.0254 (2)	0.0441 (12)	
O1	0.23874 (19)	0.7055 (6)	−0.04024 (17)	0.0497 (12)	
O2	0.15049 (19)	0.8395 (6)	−0.03665 (17)	0.0472 (11)	
O3	0.1874 (2)	0.6329 (7)	0.0914 (3)	0.0665 (16)	
O4	0.1161 (2)	0.8292 (7)	0.1003 (3)	0.0747 (16)	
Pb1	0.197071 (9)	0.95216 (3)	0.044368 (10)	0.03659 (10)	

### Atomic displacement parameters (Å^2^)

**Table:**

	*U*^11^	*U*^22^	*U*^33^	*U*^12^	*U*^13^	*U*^23^
C1	0.035 (3)	0.051 (4)	0.042 (3)	−0.010 (3)	−0.002 (3)	0.002 (3)
C2	0.068 (4)	0.047 (4)	0.051 (4)	0.012 (3)	−0.016 (3)	0.012 (3)
C3	0.077 (5)	0.076 (6)	0.051 (4)	0.001 (4)	−0.019 (4)	0.024 (4)
C4	0.075 (5)	0.086 (6)	0.049 (4)	−0.017 (5)	−0.003 (4)	−0.001 (5)
C5	0.081 (6)	0.112 (8)	0.053 (5)	0.003 (5)	−0.001 (4)	−0.029 (5)
C6	0.057 (4)	0.062 (5)	0.048 (4)	0.002 (4)	−0.002 (3)	−0.002 (4)
C7	0.041 (3)	0.044 (4)	0.037 (3)	−0.013 (3)	0.001 (3)	0.006 (3)
C8	0.042 (3)	0.044 (3)	0.031 (3)	−0.019 (3)	−0.001 (3)	0.001 (3)
C9	0.054 (4)	0.063 (4)	0.038 (3)	0.001 (4)	0.001 (3)	0.010 (3)
C10	0.075 (5)	0.081 (6)	0.069 (5)	−0.015 (5)	0.003 (4)	0.032 (5)
C11	0.061 (5)	0.096 (7)	0.083 (6)	−0.007 (5)	0.024 (4)	0.024 (5)
C12	0.040 (4)	0.062 (5)	0.077 (5)	0.000 (3)	0.014 (4)	0.008 (4)
C13	0.045 (3)	0.057 (4)	0.048 (4)	0.012 (3)	0.006 (3)	0.001 (3)
C14	0.049 (4)	0.044 (4)	0.034 (3)	−0.001 (3)	−0.007 (3)	0.008 (3)
C15	0.040 (4)	0.047 (4)	0.090 (6)	0.009 (3)	0.011 (4)	0.021 (4)
C16	0.041 (4)	0.049 (4)	0.089 (5)	0.020 (3)	0.018 (4)	0.031 (4)
C17	0.029 (3)	0.031 (3)	0.043 (3)	−0.010 (2)	−0.004 (2)	0.000 (3)
C18	0.031 (3)	0.031 (3)	0.089 (5)	0.008 (3)	0.008 (3)	0.014 (3)
C19	0.045 (4)	0.026 (3)	0.093 (5)	−0.001 (3)	0.008 (3)	0.018 (4)
Cl1	0.149 (2)	0.0533 (13)	0.1017 (18)	0.0321 (15)	−0.0395 (17)	0.0097 (13)
Cl2	0.0521 (10)	0.0578 (11)	0.0754 (12)	0.0092 (8)	0.0001 (8)	0.0201 (10)
N1	0.042 (3)	0.039 (3)	0.052 (3)	0.015 (2)	−0.003 (2)	0.001 (3)
O1	0.040 (2)	0.064 (3)	0.045 (2)	0.013 (2)	−0.0075 (19)	0.002 (2)
O2	0.041 (2)	0.051 (3)	0.049 (3)	0.013 (2)	−0.0015 (19)	−0.011 (2)
O3	0.047 (3)	0.035 (3)	0.117 (5)	0.010 (2)	0.032 (3)	0.021 (3)
O4	0.061 (3)	0.057 (3)	0.106 (4)	0.008 (3)	0.024 (3)	0.040 (3)
Pb1	0.03377 (14)	0.03153 (15)	0.04447 (15)	0.00209 (9)	−0.00447 (9)	0.00210 (10)

### Geometric parameters (Å, °)

**Table:**

C1—C6	1.344 (10)	C11—H11	0.9300
C1—C2	1.386 (9)	C12—C13	1.367 (10)
C1—C7	1.512 (8)	C12—H12	0.9300
C2—C3	1.381 (10)	C13—H13	0.9300
C2—Cl1	1.737 (8)	C14—O4	1.217 (8)
C3—C4	1.366 (12)	C14—O3	1.234 (7)
C3—H3	0.9300	C15—N1	1.284 (8)
C4—C5	1.365 (13)	C15—C16	1.373 (9)
C4—H4	0.9300	C15—H15	0.9300
C5—C6	1.400 (11)	C16—C17	1.365 (8)
C5—H5	0.9300	C16—H16	0.9300
C6—H6	0.9300	C17—C18	1.402 (8)
C7—O1	1.230 (7)	C17—C17^i^	1.467 (11)
C7—O2	1.259 (7)	C18—C19	1.383 (9)
C8—C9	1.383 (9)	C18—H18	0.9300
C8—C13	1.396 (8)	C19—N1	1.322 (8)
C8—C14	1.518 (8)	C19—H19	0.9300
C9—C10	1.364 (10)	N1—Pb1	2.523 (5)
C9—Cl2	1.764 (7)	O2—Pb1	2.301 (4)
C10—C11	1.344 (11)	O3—Pb1	2.629 (5)
C10—H10	0.9300	O4—Pb1	2.384 (5)
C11—C12	1.403 (12)		
			
C6—C1—C2	117.8 (6)	C11—C12—H12	120.0
C6—C1—C7	118.6 (6)	C12—C13—C8	121.8 (6)
C2—C1—C7	123.3 (6)	C12—C13—H13	119.1
C3—C2—C1	120.2 (7)	C8—C13—H13	119.1
C3—C2—Cl1	117.0 (5)	O4—C14—O3	122.8 (6)
C1—C2—Cl1	122.9 (5)	O4—C14—C8	116.5 (6)
C4—C3—C2	120.3 (7)	O3—C14—C8	120.6 (6)
C4—C3—H3	119.9	N1—C15—C16	123.1 (6)
C2—C3—H3	119.9	N1—C15—H15	118.4
C3—C4—C5	120.9 (8)	C16—C15—H15	118.4
C3—C4—H4	119.5	C17—C16—C15	121.3 (6)
C5—C4—H4	119.5	C17—C16—H16	119.3
C4—C5—C6	117.0 (9)	C15—C16—H16	119.3
C4—C5—H5	121.5	C16—C17—C18	115.0 (5)
C6—C5—H5	121.5	C16—C17—C17^i^	123.2 (6)
C1—C6—C5	123.6 (8)	C18—C17—C17^i^	121.7 (6)
C1—C6—H6	118.2	C19—C18—C17	119.5 (5)
C5—C6—H6	118.2	C19—C18—H18	120.2
O1—C7—O2	122.3 (6)	C17—C18—H18	120.2
O1—C7—C1	119.7 (6)	N1—C19—C18	122.5 (6)
O2—C7—C1	117.9 (5)	N1—C19—H19	118.8
C9—C8—C13	116.1 (6)	C18—C19—H19	118.8
C9—C8—C14	126.5 (6)	C15—N1—C19	118.3 (6)
C13—C8—C14	117.3 (6)	C15—N1—Pb1	117.3 (4)
C10—C9—C8	121.9 (7)	C19—N1—Pb1	124.4 (4)
C10—C9—Cl2	116.4 (6)	C7—O2—Pb1	105.9 (4)
C8—C9—Cl2	121.7 (5)	C14—O3—Pb1	87.2 (4)
C11—C10—C9	121.9 (8)	C14—O4—Pb1	99.4 (4)
C11—C10—H10	119.0	O2—Pb1—O4	88.57 (19)
C9—C10—H10	119.0	O2—Pb1—N1	75.99 (16)
C10—C11—C12	118.0 (8)	O4—Pb1—N1	77.48 (18)
C10—C11—H11	121.0	O2—Pb1—O3	88.51 (18)
C12—C11—H11	121.0	O4—Pb1—O3	50.55 (15)
C13—C12—C11	120.1 (7)	N1—Pb1—O3	126.24 (15)
C13—C12—H12	120.0		

Symmetry codes: (i) −*x*, −*y*+3, −*z*.

### Hydrogen-bond geometry (Å, °)

**Table:**

*D*—H···*A*	*D*—H	H···*A*	*D*···*A*	*D*—H···*A*
C15—H15···O1^ii^	0.93	2.40	3.239 (7)	150
C16—H16···O2^iii^	0.93	2.55	3.361 (6)	146
C19—H19···O4	0.93	2.39	3.026 (5)	125

Symmetry codes: (ii) −*x*+1/2, *y*+1/2, *z*; (iii) *x*, *y*+1, *z*.
